# Supplementary material for: Noncovalently fused-ring electron acceptors with near-infrared absorption for high-performance organic solar cells
Source: Nat Commun. 2019 Jul 10;10:3038. doi: 10.1038/s41467-019-11001-6 (PMC6620284; doi:10.1038/s41467-019-11001-6)
Supplement: Supplementary file 1 — Supplementary Information [file 41467_2019_11001_MOESM1_ESM.pdf]

SUPPLEMENTARY INFORMATION

Noncovalently Fused-Ring Electron Acceptors with Near-Infrared Absorption for High-Performance Organic Solar Cells

Hao Huang, [1] Qingxin Guo,[1] Shiyu Feng, [1] Cai'e Zhang,[1] Zhaozhao Bi, [2] Wenyue Xue,[2]Jinjin Yang, [3]Jinsheng Song,[4] Cuihong Li, [1] Xinjun Xu, [*,1] Zheng Tang,[*,3] Wei Ma[*,2] and Zhishan Bo[*,1]

[1] Key Laboratory of Energy Conversion and Storage Materials, College of Chemistry, Beijing Normal University, Beijing 100875, P. R. China.

[2] State Key Laboratory for Mechanical Behavior of Materials, Xi'an Jiaotong University, Xi'an 710049, China.

[3] Center for Advanced Low-dimension Materials, State Key Laboratory for Modification of Chemical Fibers and Polymer Materials, College of Materials Science and Engineering, Donghua University, Shanghai, 201620, P. R. China.

[4] Engineering Research Center for Nanomaterials, Henan University, Kaifeng 475004, China.

These authors contributed equally: Hao Huang, Qingxin Guo.

Correspondence and requests for materials should be addressed to X.X. (email: xuxj@bnu.edu.cn), to Z.T. (email: ztang@dhu.edu.cn), to W.M. (email: msewma@mail.xjtu.edu.cn) or to Z.B. (email: zsbo@bnu.edu.cn)

Supplementary Figures

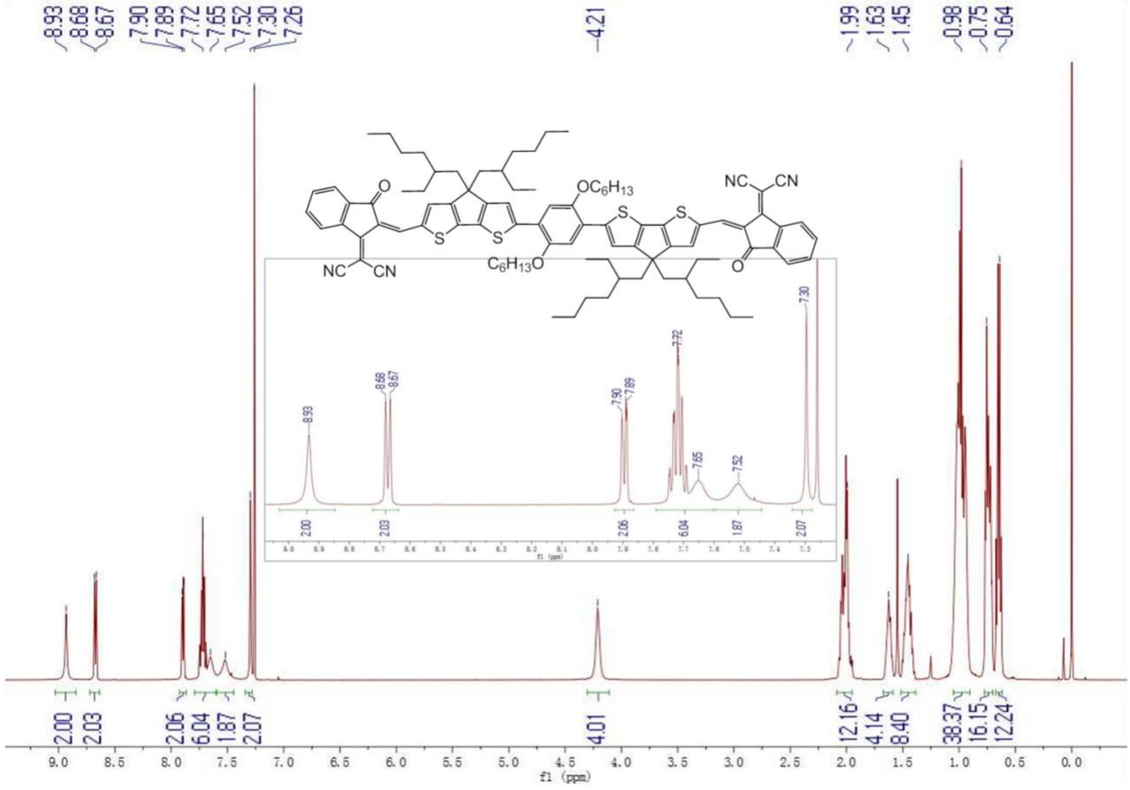

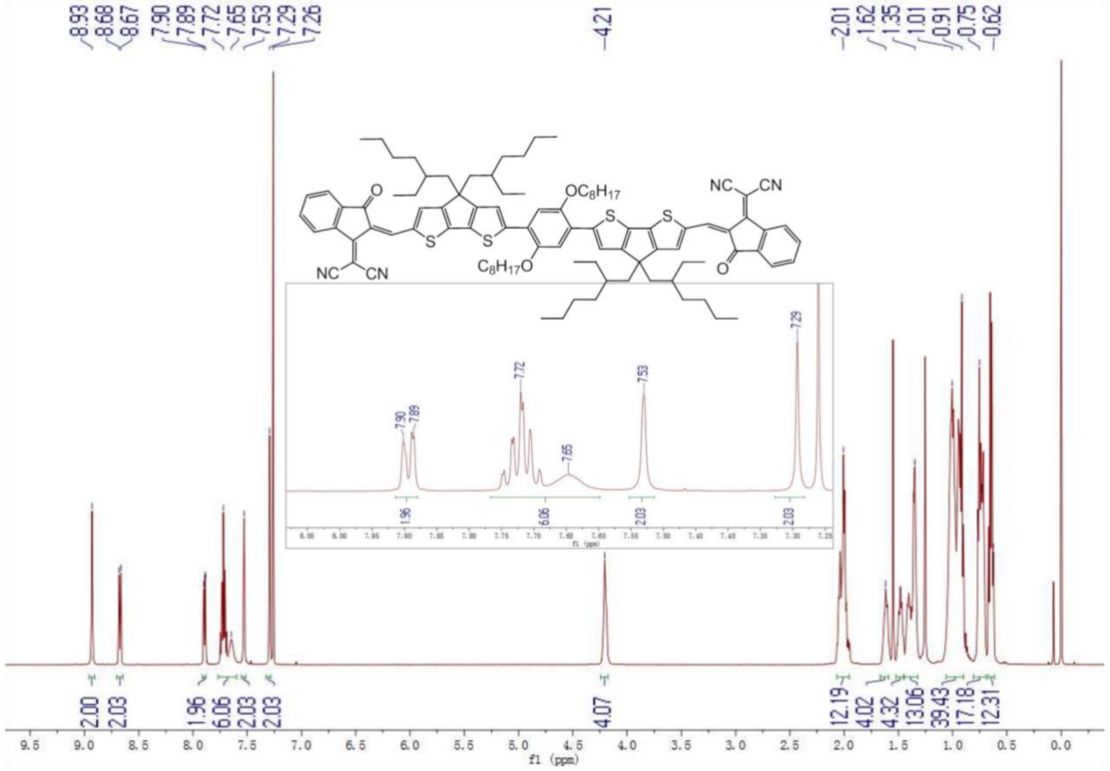

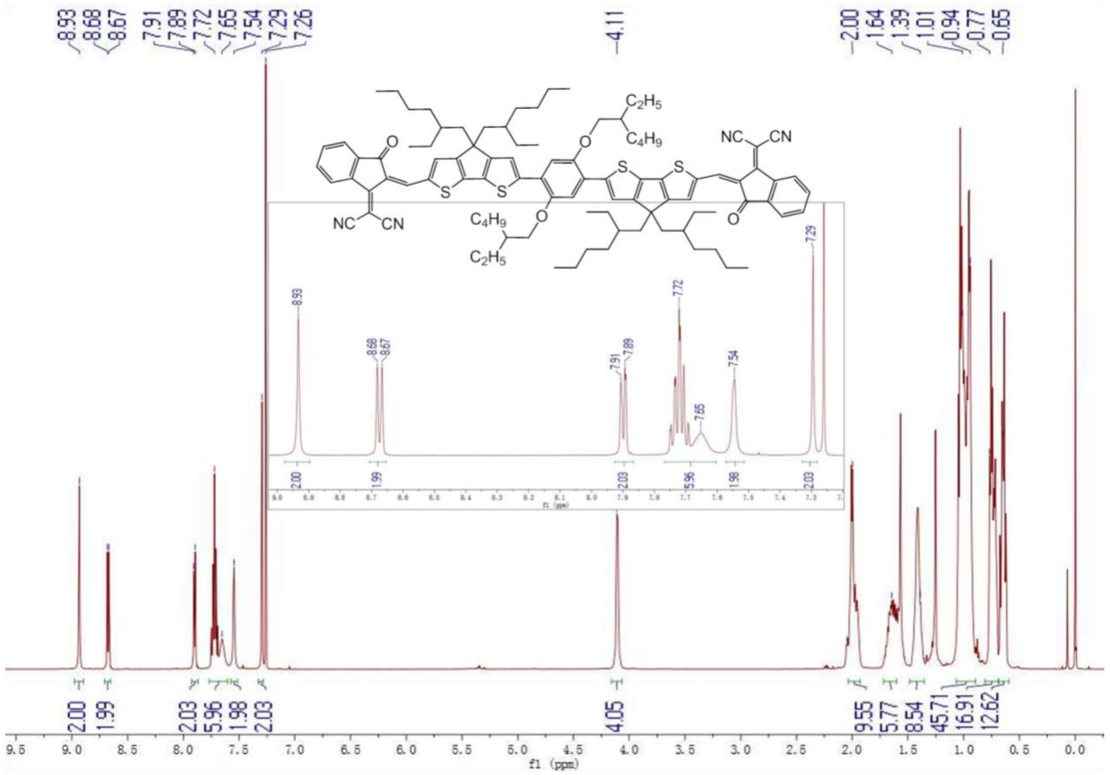
8.93
8.68
8.67
7.91
7.89
7.72
7.65
7.54
7.29
7.26
4.11
2.00
1.64
1.39
1.01
0.94
0.77
0.65
2.00
1.99
2.03
5.96
1.98
2.03
4.05
9.55
5.77
8.54
45.71
16.91
12.62
f1 (ppm)

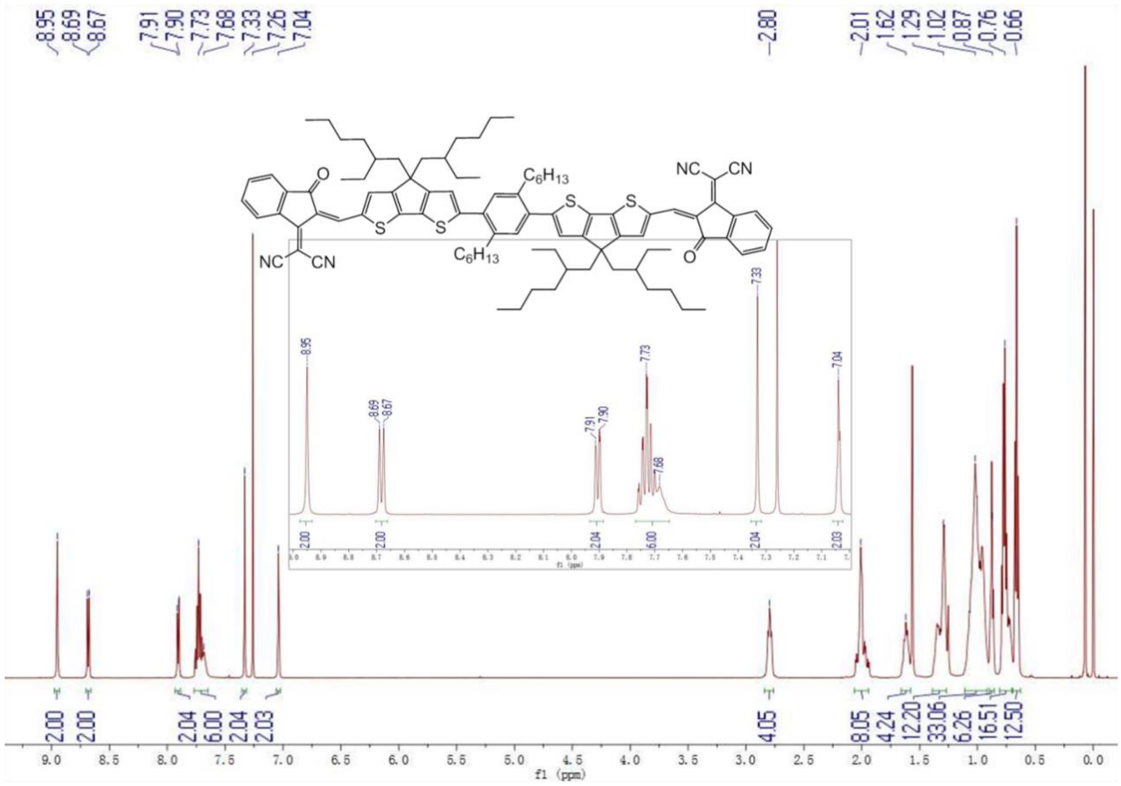
8.95
8.69
8.67
7.91
7.90
7.73
7.68
7.33
7.26
7.04
2.80
2.01
1.62
1.29
1.02
0.87
0.76
0.66
2.00
2.00
2.04
6.00
2.04
2.03
4.05
8.05
4.24
12.20
33.06
6.26
16.51
12.50
f1 (ppm)

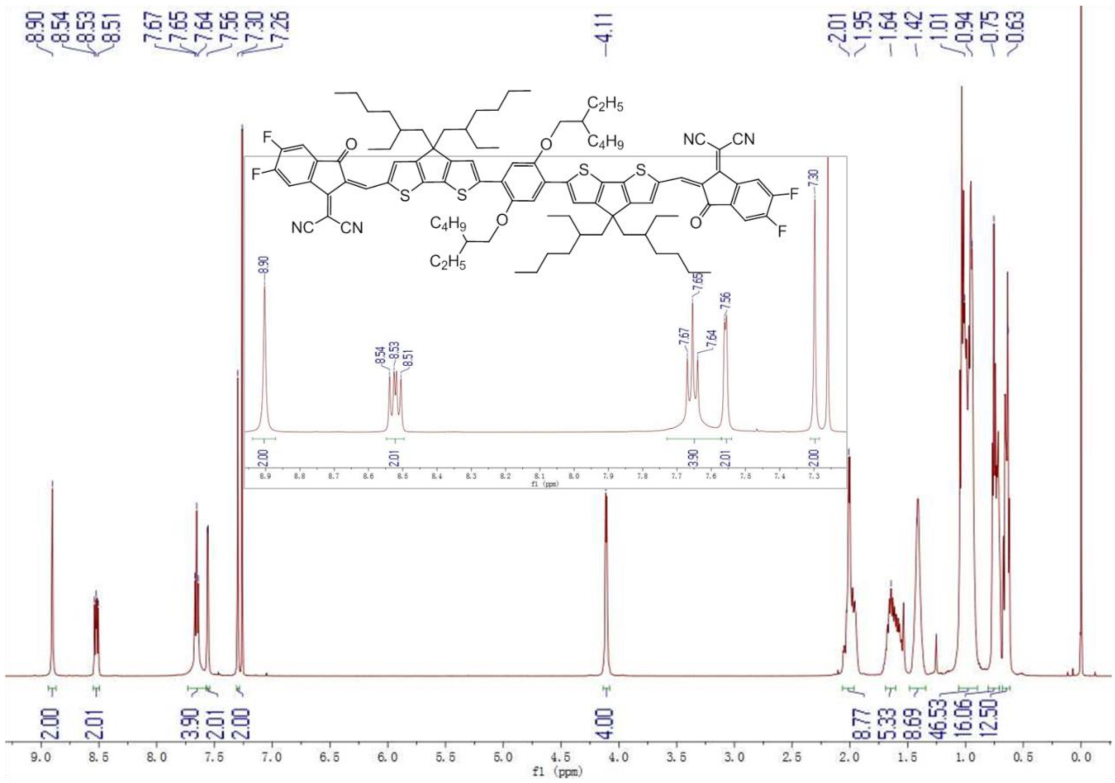

Supplementary Figure 1. ^{1}H NMR spectra of DOC6-IC, DOC8-IC, DOC2C6-IC, DC6-IC and DOC2C6-2F.

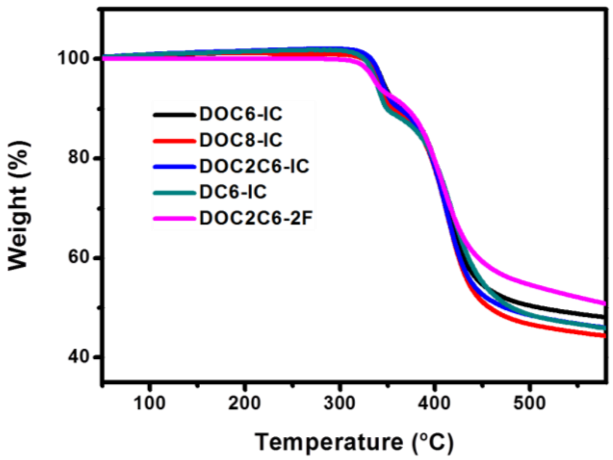

Supplementary Figure 2. TGA curves of DOC6-IC, DOC8-IC, DOC2C6-IC, DC6-IC and DOC2C6-2F under nitrogen atmosphere at heating rate of 10 °C min^{-1}. Source data are provided as a Source Data file.

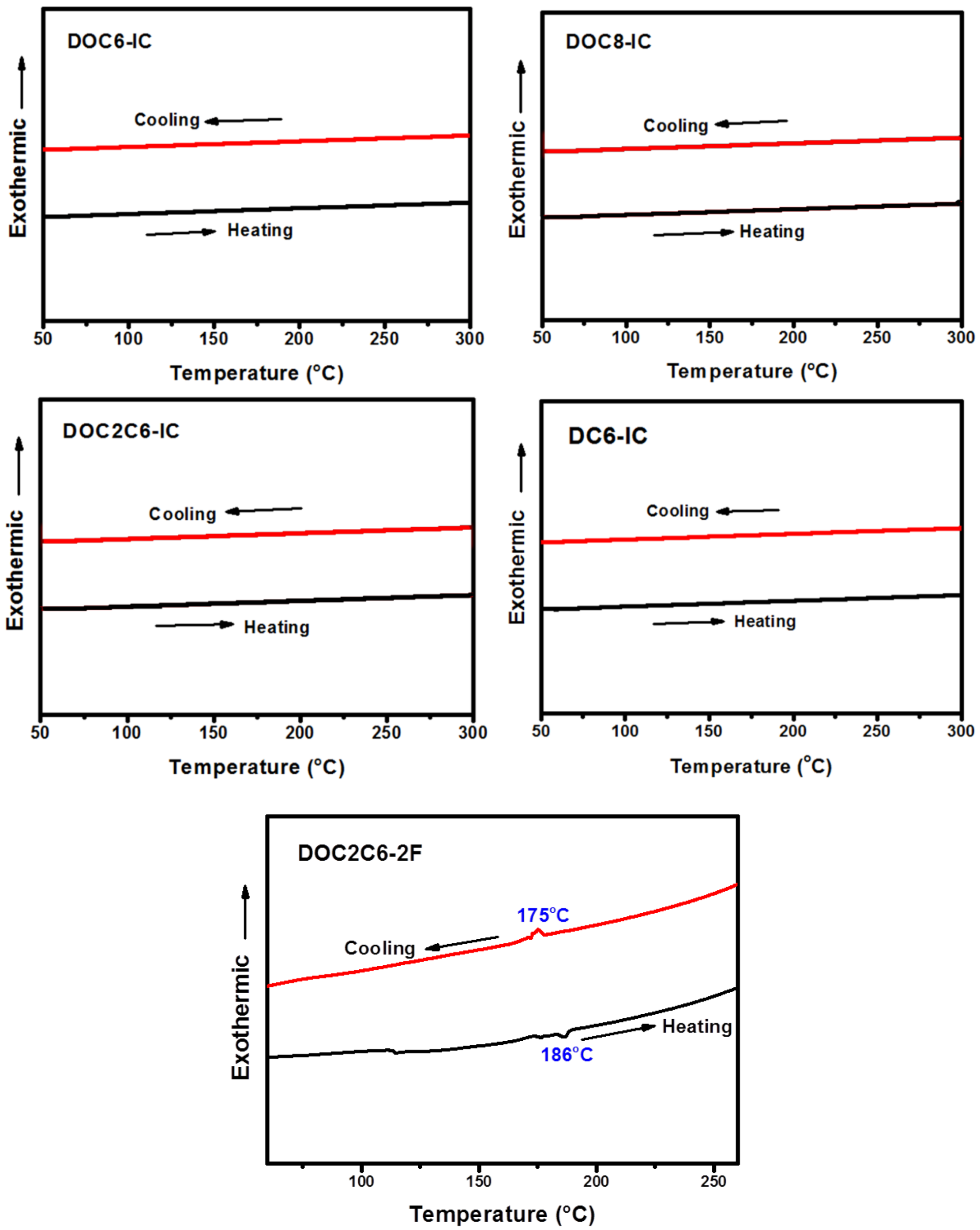

Supplementary Figure 3. DSC curves of DOC6-IC, DOC8-IC, DOC2C6-IC, DC6-IC and DOC2C6-2F under nitrogen atmosphere at heating and cooling rate of 10 °C min^{-1}. Source data are provided as a Source Data file.

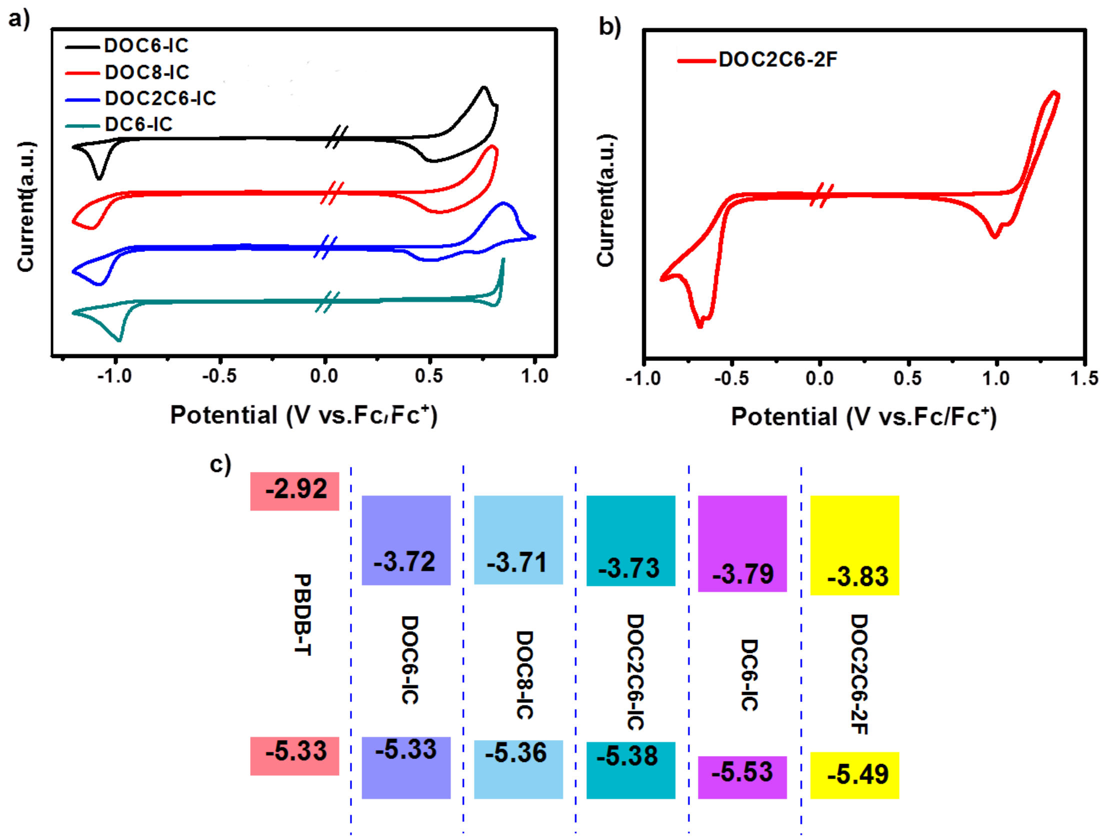

Supplementary Figure 4. **a)** Cyclic voltammograms of DOC6-IC, DOC8-IC, DOC2C6-IC and DC6-IC as films and **b)** Cyclic voltammograms of DOC2C6-2F as film and **c)** energy level diagrams. Source data are provided as a Source Data file.

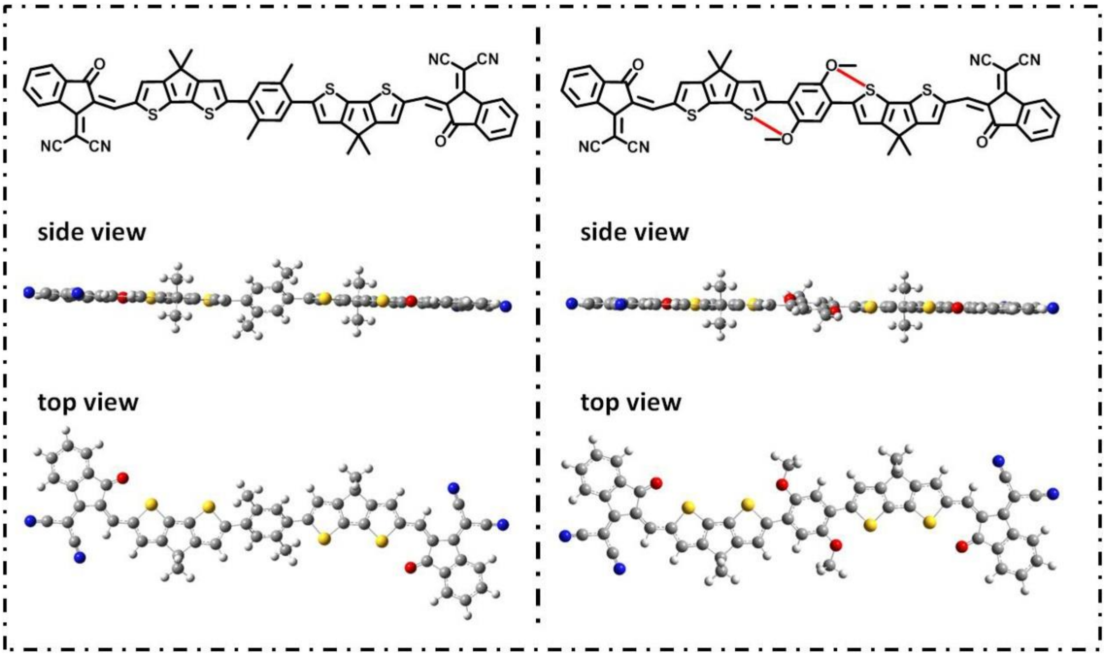

Supplementary Figure 5. Simulated molecular geometries by DFT calculations for simplified molecules of DC6-IC and DOC-ICs.

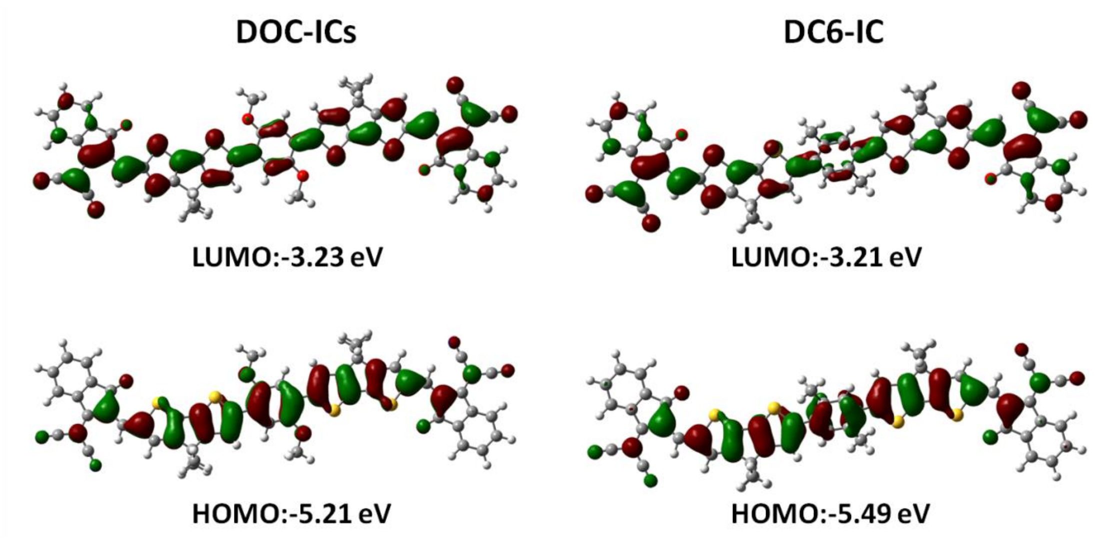

Supplementary Figure 6. Frontier molecular orbitals calculated by density functional theory.

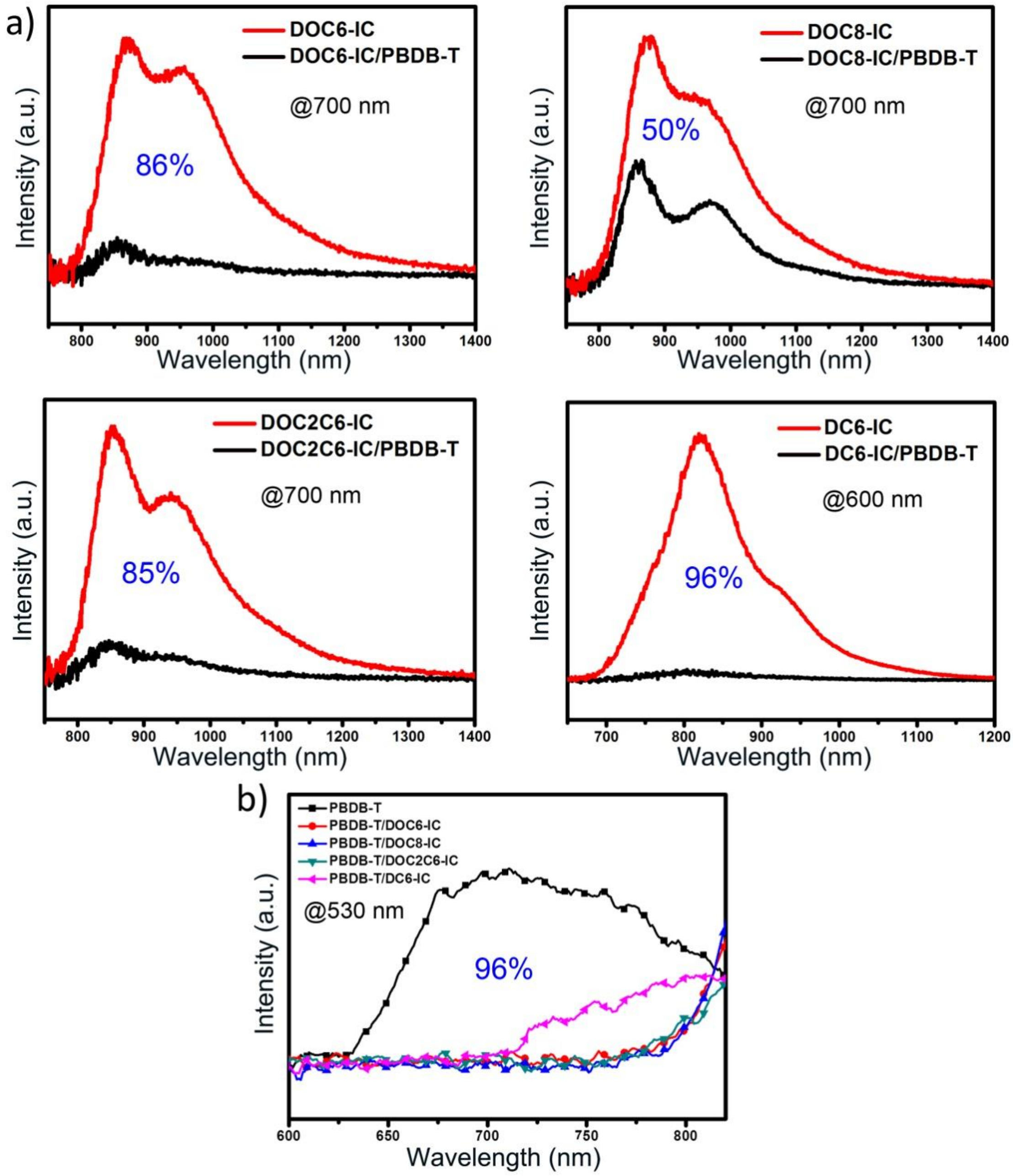

Supplementary Figure 7. Fluorescence emission spectra of **a)** DOC6-IC, DOC8-IC, DOC2C6-IC and DC6-IC and PBDB-T:DOC6/DOC8/DOC2C6/DC6-IC films and **b)** neat film of PBDB-T and PBDB-T:DOC6/DOC8/DOC2C6/DC6-IC films. Source data are provided as a Source Data file.

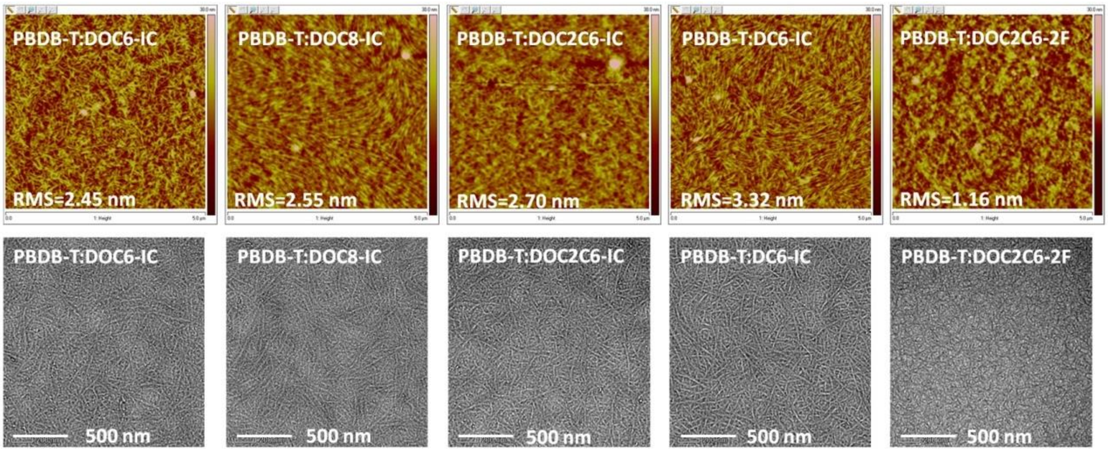

Supplementary Figure 8. AFM and TEM images of PBDB-T:DOC6/DOC8/DOC2C6/DC6-IC and PBDB-T:DOC2C6 -2F blend films.

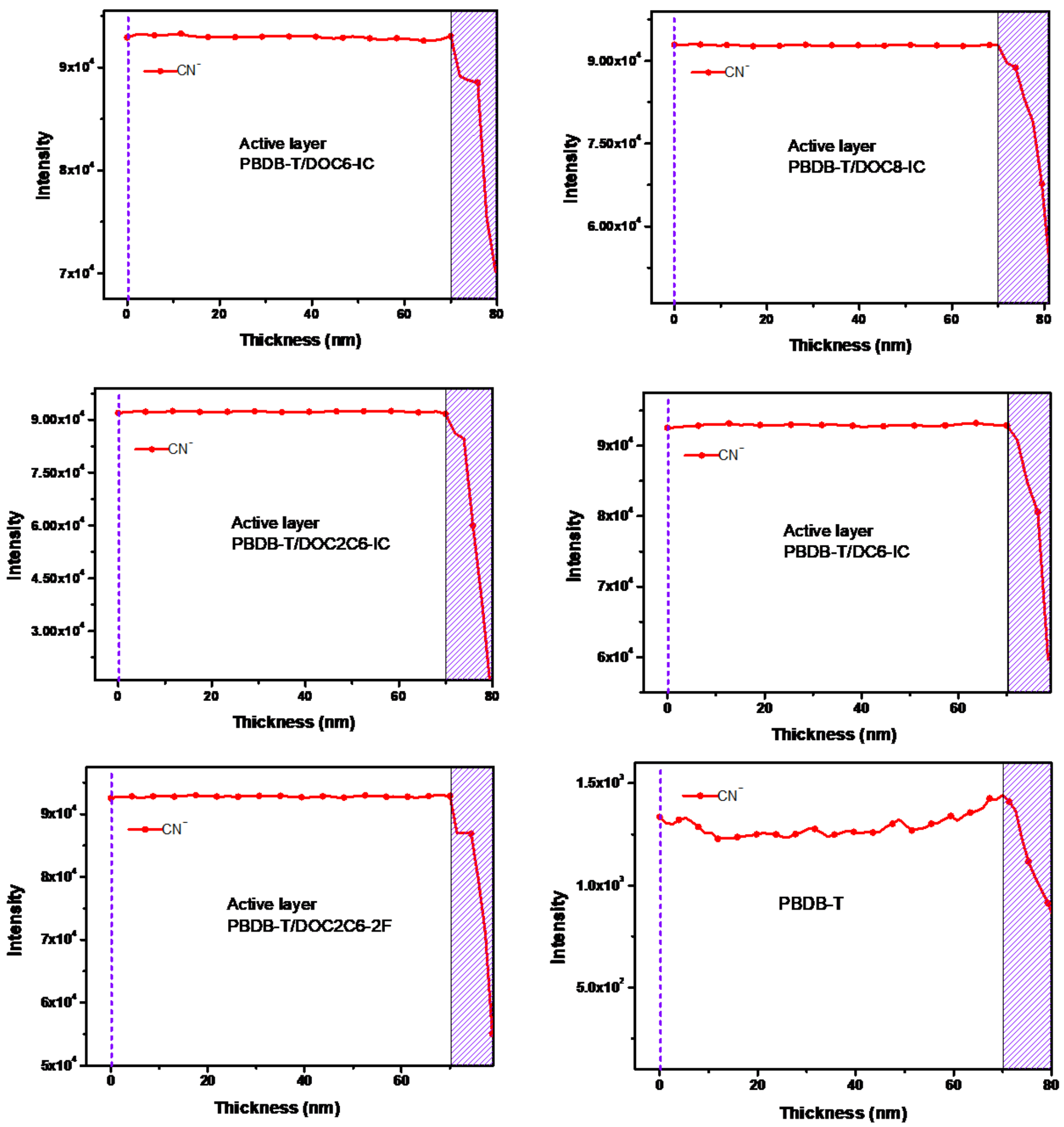

Supplementary Figure 9. The intensity of CN^- in active layers and PBDB-T measured by ToF-SIMS. Source data are provided as a Source Data file.

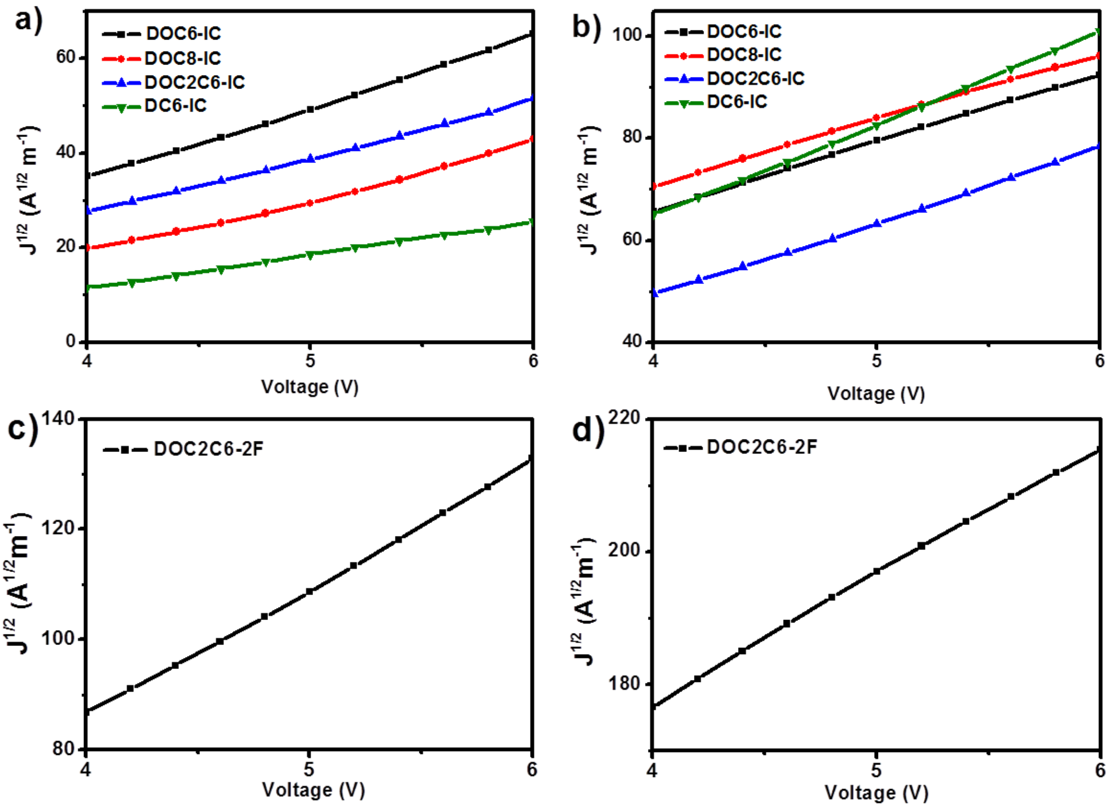

Supplementary Figure 10. SCLC measurements for determining **a)** the electron and **b)** hole mobilities of PBDB-T:DOC6/DOC8/DOC2C6/DC6-IC blends; **c)** the electron and **d)** hole mobilities of PBDB-T:DOC2C6 -2F blend. Source data are provided as a Source Data file.

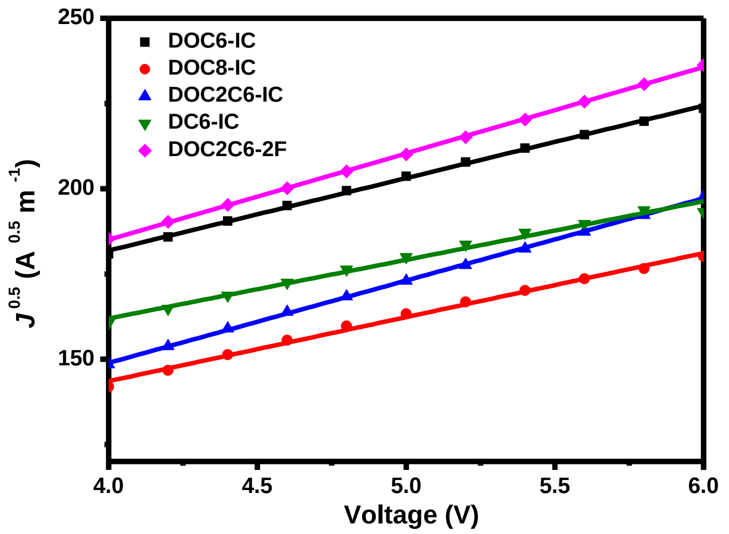

Supplementary Figure 11. SCLC measurements for determining mobilities of neat acceptor films. Source data are provided as a Source Data file.

Supplementary Tables

Supplementary Table 1. Optical and electrochemical properties of DOC6-IC, DOC8-IC, DOC2C6-IC, DC6-IC and DOC2C6-2F

Compounds	$\lambda^{a}_{\mathrm{max}}$[nm] sol.	$\lambda^{b}_{\mathrm{max}}$[nm] film	ε^{c} [M^{-1} cm^{-1}]	$E^{d}_{\mathrm{g.opt}}$ [eV]	HOMO[e][eV]/ LUMO[f][eV]	$E_{\mathrm{g.opt}}/q-V_{\mathrm{oc}}$ [V]	E^{g}_{U} [eV]
DOC6-IC	722	714,778	2.05×10^{5}	1.43	–5.33/–3.72	0.52	0.027
DOC8-IC	724	714,778	2.22×10^{5}	1.39	–5.36/–3.71	0.47	0.025
DOC2C6-IC	720	709,764	2.06×10^{5}	1.44	–5.38/–3.73	0.51	0.036
DC6-IC	623	634	1.91×10^{5}	1.69	–5.53/–3.79	0.70	0.072
DOC2C6-2F	743	717,780	1.75×10^{5}	1.42	–5.49/–3.83	0.57	0.026

[a] Absorption maximum in solution; [b] absorption maximum in film; [c] molar extinction coefficient at λ_{max} in solution; [d] optical bandgap calculated from the film absorption onsets, Determining the optical bandgap by the intersection of the linear fitting curve of the absorption spectrum and the tangent of absorption tail; [e] HOMO energy levels estimated from the onset oxidation potentials; [f] LUMO energy levels estimated from the onset reduction potentials; [g] Urbach energy of the molecular acceptors calculated using the method described in reference.[1]

Supplementary Table 2. Photovoltaic performance of devices fabricated with PBDB-T and DOC6-IC.

System	D/A [w/w]	Thickness	V_{oc} [V]	J_{sc} [mA/cm^2]	*FF*	PCE$_{max}$ [%]
PBDB-T/ DOC6-IC	1:0.6	100 nm	0.92	14.84	52.38	7.16
	1:0.8		0.91	16.90	55.88	8.72
	1:1		0.91	15.53	48.31	6.85
	1:1.2		0.91	13.95	46.16	5.86

Supplementary Table 3. Photovoltaic performance of devices fabricated with PBDB-T and DOC6-IC.

System	D/A [w/w]	Thickness	V_{oc} [V]	J_{sc}[mA/cm^2]	*FF*	PCE$_{max}$ [%]
PBDB-T/ DOC6-IC	1:0.8	100-110 nm	0.91	16.90	55.88	8.72
		60-70 nm	0.91	19.21	60.11	10.52

Supplementary Table 4. Photovoltaic performance of devices fabricated with PBDB-T and DOC2C6-2F.

System	D/A [w/w]	annealing [°C]	V_{oc} [V]	J_{sc} [mA/cm^2]	*FF*	PCE$_{max}$ [%]
PBDB-T/	1:0.8		0.87	19.23	70.45	11.69
DOC2C6-2F		100	0.85	21.35	73.15	13.24

Supplementary Table 5. Structural parameters obtained by fitting the out-of-plane profiles from GIWAXS.

	D-spacing/Å	C.L./Å
PBDB-T/DOC6-IC	3.7	33.2
PBDB-T/DOC8-IC	3.7	32.8
PBDB-T/DOC2C6-IC	3.7	31.4
PBDB-T/DC6-IC	3.7	28.6
PBDB-T/DOC2C6-2F	3.6	44.8
DOC6-IC	3.7	27.0
DOC8-IC	3.7	21.7
DOC2C6-IC	3.7	20.6
DOC2C6-2F	3.8	32.8

Supplementary Table 6. Structural parameters obtained by fitting the in-plane profiles from GIWAXS.

	D-spacing/Å	C.L./Å
PBDB-T/DOC6-IC	21.3	155.1
	17.6	74.3
PBDB-T/DOC8-IC	21.2	156.4
	18.5	120.2
PBDB-T/DOC2C6-IC	21.0	147.4
PBDB-T/DC6-IC	21.1	145.3
PBDB-T/DOC2C6-2F	27.3	92.0
	17.0	138.7
DOC2C6-2F	21.1	267.1
	16.1	128.8

Supplementary Table 7. Contact angle of water and glycerol and surface tension of DOC6-IC, DOC8-IC, DOC2C6-IC, DC6-IC, DOC2C6-2F, and PBDB-T.

	ϑ_{water} [deg]	ϑ_{GL} [deg]	γ [mJ·m^{-1}]
DOC6-IC	99.3	85.4	23.49
DOC8-IC	101.4	85.5	23.81
DOC2C6-IC	99.5	86	23.15
DC6-IC	98.2	91.1	21.77
DOC2C6-2F	99.3	86.6	22.85
PBDB-T	104.1	92	20.17

Supplementary Table 8. Flory-Huggins interaction parameters for acceptors and donor.

Active layers	χ
DOC6-IC/PBDB-T	0.42
DOC8-IC/PBDB-T	0.44
DOC2C6-IC/PBDB-T	0.41
DC6-IC/PBDB-T	0.36
DOC2C6-2F/PBDB-T	0.39

Supplementary Table 9. The electron mobility and hole mobility of PBDB-T:DOC6/DOC8/DOC2C6/DC6-IC blends and PBDB-T:DOC2C6-2F blend.

System	μ_e (cm^2 V^{-1} s^{-1})	μ_h (cm^2 V^{-1} s^{-1})
PBDB-T/DOC6-IC	3.59×10^{-4}	2.86×10^{-4}
PBDB-T/DOC8-IC	2.09×10^{-4}	2.63×10^{-4}
PBDB-T/DOC2C6-IC	2.22×10^{-4}	3.33×10^{-4}
PBDB-T/DC6-IC	7.88×10^{-5}	5.15×10^{-4}
PBDB-T/DOC2C6-2F	2.44×10^{-3}	1.74×10^{-3}

Supplementary Table 10. Mobilities in neat films measured by SCLC method.

System	μ (cm^2 V^{-1} s^{-1})
DOC6-IC	2.08×10^{-3}
DOC8-IC	1.63×10^{-3}
DOC2C6-IC	2.69×10^{-3}
DC6-IC	1.36×10^{-3}
DOC2C6-2F	2.96×10^{-3}

Supplementary Notes

Supplementary Note 1. Thermogravimetric analysis (TGA) and differential scanning calorimetry (DSC) Measurements: Thermogravimetric analysis (TGA) indicates that all these small molecules have a good thermal stability with decomposition temperatures (5% weight loss) up to approximately 350 °C (see Supplementary Figure 2) under nitrogen atmosphere. And the differential scanning calorimetry (DSC) measurements shown in Supplementary Figure 3 demonstrated there is no obvious glass transition, crystallization or melting peak in the range of 50 to 300 °C under a heating or cooling rate of 10 °C min^{-1} for DOC6-IC, DOC8-IC, DOC2C6-IC and DC6-IC, indicating these compounds have a weak tendency to form large size crystalline domains. But the DSC measurement shows a crystallization peak at 175 °C at a cooling rate of 10 °C min^{-1} and a melting peak at 186 °C at a heating rate of 10 °C min^{-1} for DOC2C6-2F, indicating that fluorination can promote the aggregation of molecules.

Supplementary Note 2. Cyclic voltammetry (CV) measurements: Cyclic voltammetry (CV) measurements (see Supplementary Figure 4) show that the film onset oxidation and reduction potentials are 0.62 and –0.99 V for DOC6-IC, 0.65 and –1.00 V for DOC8-IC, 0.67 and –0.98 V for DOC2C6-IC, 0.82 and –0.92 V for DC6-IC film, respectively, with a Pt wire counter electrode, and an $Ag/AgNO_3$ (0.01 M in CH_3CN) reference electrode. The highest occupied molecular orbital (HOMO) and lowest unoccupied molecular orbital (LUMO) levels of DC6-IC are calculated to be -5.53 and -3.79 eV, respectively, according to the equation[2]

$$E = -e[E_{ox/red} + 4.80 - E_{(Fc/Fc+)}] \quad (1)$$

As for DOC-ICs, the introduction of electron-donating 2,5-bis(alkyloxy)phenylene unit could raise the HOMO and LUMO levels for approximate 0.15 to 0.20 eV and 0.06 to 0.08 eV, respectively. CV measurements show that the film onset oxidation and reduction potentials are 1.13 and –0.53 V for DOC2C6-2F film, respectively, with a Pt wire counter electrode and an $Ag/AgCl/Cl^-$ reference electrode and the HOMO and LUMO levels of DOC2C6-2F are calculated to be -5.49 and -3.83 eV, respectively. All these data are summarized in Supplementary Table 4.

Supplementary Note 3. Density functional theory (DFT) calculations: Density functional theory (DFT) calculations are also performed to investigate the chemical geometry of DOC-ICs and DC6-IC with simplified side chains, as shown in Supplementary Figure 5. The dihedral angles between the central phenylene unit and the two neighboring 4H-cyclopenta[2,1-b:3,4-b']dithiophene units are 9.4° for DOC-ICs and 40.9° for DC6-IC. The large difference of dihedral angles also indicate that the introduction of intramolecular O···S interaction in DOC-ICs could reduce the rotation of single bond in the molecules in contrast to DC6-IC.

Supplementary Note 4. Space-charge limited current measurement: Hole/electron devices with a structure of ITO/PEDOT:PSS (30 nm)/active layer (80 nm)/Au and ITO/ZnO (30 nm)/active layer (80 nm)/Ag (100 nm) are fabricated. Hole and electron mobilities of devices are calculated

according to the Mott-Gurney equation:

$$J = 9\varepsilon_o\varepsilon_r\mu V^2/8d^3 \quad (2)$$

where J is the space charge limited current, ε_o is the vacuum permittivity (ε_o = 8.85×10^{-12} F/m), ε_r is the permittivity of the active layer (ε_r = 3), μ is mobility, and d is the thickness of the active layer.

Supplementary Note 5. Calculation of Flory-Huggins interaction parameters: The surface energy γ values could be calculated according to the Wu model on the neat films by the Equation:[3]

$$\gamma = \gamma^{\mathrm{d}} + \gamma^{\mathrm{p}} \quad (3)$$

$$\gamma_{\mathrm{LV}}(1+\cos\theta) = \frac{4\gamma_{\mathrm{S}}^{\mathrm{d}}\gamma_{\mathrm{L}}^{\mathrm{d}}}{\gamma_{\mathrm{S}}^{\mathrm{d}}+\gamma_{\mathrm{L}}^{\mathrm{d}}} + \frac{4\gamma_{\mathrm{S}}^{\mathrm{p}}\gamma_{\mathrm{L}}^{\mathrm{p}}}{\gamma_{\mathrm{S}}^{\mathrm{p}}+\gamma_{\mathrm{L}}^{\mathrm{p}}} \quad (4)$$

And the two different contact angles of water and glycerol are measured to achieve the γ of acceptor and polymer donor, and the results are shown in Supplementary Table 5. And the γ is the sum of dispersion (d) and polar (p) components.

As $\delta \propto \sqrt{\gamma}$, we could calculate all the solubility parameter (δ) of acceptor and polymer.[4] Further, the Flory-Huggins interaction parameter χ could be calculated according to the Equation:[4]

$$\chi_{ij} = \frac{V_1}{RT}\left(\delta_i - \delta_j\right)^2 + 0.34 \quad (5)$$

Since we adopt the *o*-DCB as the solvent, the V_1 is 113.3 cm^3 mol^{-1}. We calculate the χ of the blend of small molecular acceptors and PBDB-T, and the results are shown in Supplementary Table 6.

Supplementary Methods

Material and instruments: Unless otherwise specified, all chemicals were purchased from Aldrich, Acros or Derthon and used without further purification. The catalyst precursor $Pd(PPh_3)_4$ was prepared according to the literature and stored in a Schlenk tube under nitrogen atmosphere. Unless otherwise specified, all reactions were performed under an atmosphere of nitrogen and monitored by thin layer chromatography (TLC) on silica gel column chromatography was carried out on silica gel (200-300 mesh). ^1H and ^{13}C NMR spectra were recorded on a Bruker AV 400 or 600 spectrometer. UV-visible absorption spectra were obtained on a PerkinElmer UV-vis spectrometer model Lambda 750. Thermogravimetric analysis (TGA) measurements were performed on TA2100 at a heating rate of 10 °C min^{-1} from 40 °C to 600 °C. Differential scanning calorimetry (DSC) measurements were tested on Perkin-Elmer Diamond DSC instrument, under a nitrogen atmosphere at a heating rate and cool rate of 10 °C min^{-1} from 50 °C to 300 °C. The electrochemical behavior of the polymers was investigated using cyclic voltammetry (CHI 630A Electrochemical Analyzer) with a standard three-electrode electrochemical cell in a 0.1 M Bu_4NPF_6 solution in CH_3CN at room temperature under an atmosphere of nitrogen with a scanning rate of 100 mV s^{-1}. A Pt plate working electrode, a Pt wire counter electrode, and an $Ag/AgNO_3$ (0.01 M in CH_3CN) reference electrode or an $Ag/AgCl/Cl^-$ reference electrode were used. The experiments were calibrated with the standard ferrocene/ferrocenium (Fc) redox system and assumption that the energy level of Fc is 4.8 eV below vacuum. MS (MALDI-TOF) measurements were performed with an Autoflex III instrument. The absolute fluorescence quantum yields were tested with Edinburgh FLS980 Spectrometer under the Xenon source light path and NIRPMT-sphere detector light path. Grazing Incidence Wide-Angle X-ray Scattering (GIWAXS) measurements were performed at beamline 7.3.3 at the Advanced Light Source.[5] Samples were prepared on Si substrates using identical blend solutions as those used in devices. The 10 keV X-ray beam was incident at a grazing angle of 0.12°-0.16°, selected to maximize the scattering intensity from the samples. The scattered x-rays were detected using a Dectris Pilatus 2M photon counting detector.

Synthetic procedures and characterization:

(4,4-Bis(2-ethylhexyl)-4H-cyclopenta[2,1-b:3,4-b']dithiophen-2-yl)trimethylstannane (2)

To a solution of 1 (500 mg, 1.24 mmol) in 10 mL THF (tetrahydrofuran), *n*-BuLi (0.62 mL, 2.4 M, 1.49 mmol) was added dropwise under the protection of nitrogen at -78 °C. The mixture was allowed to warm to 0 °C slowly. After the mixture was stirred at 0 °C for 2 h, trimethyltin chloride (1.48 mL, 1.0 M, 1.49 mmol) was added in one portion. And the mixture was stirred overnight and allowed to warm to room temperature gradually. The solvent was evaporated to dryness under vacuum to afford 2 as a dark orange oil in a yield of 96%, and the crude product was used directly in the next reaction without further purification.

^{1}H NMR (600 MHz, $CDCl_3$) δ 7.08 (d, J = 4.9 Hz, 1H), 6.97-6.88 (m, 2H), 1.91-1.79 (m, 4H), 1.03-0.81 (m, 16H), 0.74 (q, J = 6.6 Hz, 6H), 0.62-0.55 (m, 8H), 0.36 (s, 9H).

2,2'-(2,5-Bis(hexyloxy)-1,4-phenylene)bis(4,4-bis(2-ethylhexyl)-4H-cyclopenta[1,2-b:5,4-b']

dithiophene) (4a)

A mixture of 2 (552 mg, 0.98 mmol) and 1,4-dibromo-2,5-bis(hexyloxy)benzene (170 mg, 0.39 mmol) in toluene was degassed before and after $Pd(PPh_3)_4$ (45 mg) was added, the mixture was refluxed under N_2 for 60 hours. After the reaction was finished, the mixture was poured into water, extracted by DCM. The mixture was chromatographically purified on silica gel column with DCM/petroleum ether (1:12, v/v) to give 4a as an orange oil in a yield of 90% (380 mg).

^{1}H NMR (600 MHz, $CDCl_3$) δ 7.41 (s, 2H), 7.21 (s, 2H), 7.12 (d, *J*=4.8 Hz, 2H), 6.93 (d, *J*=4.68 Hz, 2H), 4.12 (t, *J*=6.28 Hz, 4H), 1.98-1.82 (m, 12H), 1.64-1.53 (m, 4H), 1.42-1.39 (m, 8H), 1.05-0.89 (m, 38H), 0.78-0.69 (m, 16H), 0.61-0.55 (m, 12H).

^{13}C NMR (125 MHz, $CDCl_3$) δ 158.00, 157.80, 149.65, 139.50, 137.92, 137.81, 124.59, 123.84, 122.80, 120.87, 112.43, 70.23, 53.95, 43.84, 35.53, 34.69, 34.64, 32.17, 29.98, 29.10, 27.83, 26.52, 23.28, 23.16, 14.58, 11.14.

MS (MALDI-TOF): Calcd for $C_{68}H_{102}O_2S_4$ (M^+): 1078.68, Found: 1078.85.

2,2'-(2,5-Bis(octyloxy)-1,4-phenylene)bis(4,4-bis(2-ethylhexyl)-4H-cyclopenta[1,2-b:5,4-b'] dithiophene) (4b)

A mixture of 2 (384.54 mg, 0.68 mmol) and1,4-dibromo-2,5-bis(octyloxy)benzene (154 mg, 0.31 mmol) in toluene was degassed before and after $Pd(PPh_3)_4$ (36 mg) was added, the mixture was refluxed under N_2 for 60 hours. After the reaction was finished, the mixture was poured into water, extracted by DCM. The mixture was chromatographically purified on silica gel column with DCM/petroleum ether (1:15, v/v) to give 4b as an orange oil in a yield of 80% (282 mg).

^{1}H NMR (400 MHz, $CDCl_3$) δ 7.42 (s, 2H), 7.21 (s, 2H), 7.13 (d, *J*=4.88 Hz, 2H), 6.95-6.93 (m, 2H), 4.13 (t, *J*=6.48 Hz, 4H), 1.98-1.85 (m, 12H), 1.62-1.54 (m, 4H), 1.46-1.25 (m, 8H), 1.06-0.83 (m, 38H), 0.79-0.70 (m, 16H), 0.63-0.60 (m, 12H).

^{13}C NMR (125 MHz, $CDCl_3$) δ 158.02, 157.80, 149.66, 139.48, 137.88, 137.81, 124.60, 123.87, 122.84, 121.00, 112.51, 70.23, 53.95, 43.85, 35.52, 34.69, 34.61, 32.38, 30.02, 29.96, 29.83, 29.10, 27.83, 26.88, 23.28, 23.19, 14.58, 11.15.

MS (MALDI-TOF): Calcd for $C_{72}H_{110}O_2S_4$ (M^+): 1134.74, Found: 1134.93.

2,2'-(2,5-Bis((2-ethylhexyl)oxy)-1,4-phenylene)bis(4,4-bis(2-ethylhexyl)-4H-cyclopenta[1,2-b: 5,4-b']dithiophene) (4c)

A mixture of 2 (608 mg, 1.01 mmol) and 1,4-dibromo-2,5-bis((2-ethylhexyl)oxy)benzene (210 mg, 0.43 mmol) in toluene was degassed before and after $Pd(PPh_3)_4$ (50 mg) was added, the mixture was refluxed under N_2 for 60 hours. After the reaction was finished, the mixture was poured into water, extracted by DCM. The mixture was chromatographically purified on silica gel column with DCM/petroleum ether (1:20, v/v) to give 4c as an orange oil in a yield of 67% (320 mg).

^{1}H NMR (600 MHz, $CDCl_3$) δ 7.44 (s, 2H), 7.21 (s, 2H), 7.13 (d, *J*=4.44 Hz, 2H), 6.95-6.93 (m, 2H), 4.06-3.96 (m, 4H), 1.94-1.85 (m, 8H), 1.67-1.56 (m, 4H), 1.41-1.30 (m, 8H), 1.07-0.88 (m, 46H), 0.77-0.69 (m, 16H), 0.65-0.60 (m, 12H).

^{13}C NMR (125 MHz, $CDCl_3$) δ 158.14, 157.76, 149.76, 139.84, 137.84, 137.59, 124.59, 123.65, 122.87, 121.31, 112.06, 72.11, 53.98, 43.94, 40.25, 35.60, 34.71, 34.58, 31.32, 29.75, 29.08,

27.87, 24.66, 23.65, 23.29, 14.69, 11.75, 11.15.

MS (MALDI-TOF): Calcd for $C_{72}H_{110}O_2S_4$ (M^+): 1134.74, Found: 1134.93.

2,2'-(2,5-Dihexyl-1,4-phenylene)bis(4,4-bis(2-ethylhexyl)-4H-cyclopenta[1,2-b:5,4-b'] dithiophene) (4d)

A mixture of 2 (608 mg, 1.01 mmol) and 1,4-dibromo-2,5-dihexylbenzene (172 mg, 0.43 mmol) in toluene was degassed before and after $Pd(PPh_3)_4$ (50 mg) was added, the mixture was refluxed under N_2 for 60 hours. After the reaction was finished, the mixture was poured into water, extracted by DCM. The residue was chromatographically purified on silica gel column with petroleum ether to give 4d as a dark yellow oil in a yield of 61% (270 mg).

^{1}H NMR (600 MHz, $CDCl_3$) δ 7.29 (s, 2H), 7.14 (d, *J*=4.80 Hz, 2H), 6.95-6.93 (m, 2H), 6.91-6.90 (m, 2H), 2.81-2.75 (m, 4H), 1.96-1.86 (m, 8H), 1.64-1.57 (m, 4H), 1.37-1.25 (m, 12H), 1.09-0.84 (m, 38H), 0.79-0.74 (m, 12H), 0.71-0.60 (m, 16H).

^{13}C NMR (125 MHz, $CDCl_3$) δ 157.89, 157.48, 142.32, 138.83, 137.56, 137.31, 134.46, 132.65, 124.56, 122.72, 122.42, 54.13, 43.81, 35.59, 34.88, 33.73, 32.22, 29.84, 29.24, 29.13, 27.94, 23.39, 23.27, 23.12, 14.57, 11.30.

MS (MALDI-TOF): Calcd for $C_{68}H_{102}S_4$ (M^+): 1046.69, Found: 1046.78.

6,6'-(2,5-Bis(hexyloxy)-1,4-phenylene)bis(4,4-bis(2-ethylhexyl)-4H-cyclopenta[1,2-b:5,4-b'] dithiophene-2-carbaldehyde) (5a)

To a solution of 4a (380 mg, 0.35 mmol) in THF (30 mL), *n*-BuLi (0.88 mL, 2.4 M, 2.1 mmol) was added dropwise under the protection of nitrogen at -78 °C. After the mixture was stirred for 1 h at this temperature, DMF (1 mL) was added in one portion. Then the mixture was warmed to room temperature and stirred overnight. The mixture was poured into water, extracted by ethyl acetate and the obtained product was chromatographically purified on silica gel column with ethyl acetate/petroleum ether (1:12, v/v) to give 5a as an orange oil in a yield of 86% (344 mg).

^{1}H NMR (600 MHz, $CDCl_3$) δ 9.83 (s, 2H), 7.57 (d, *J*=5.46 Hz, 2H), 7.47 (d, *J*=2.28 Hz, 2H), 7.25 (d, 2H), 4.16 (t, *J*=6.54 Hz, 4H), 2.02-1.91 (m, 12H), 1.62-1.57 (m, 4H), 1.45-1.37 (m, 8H), 1.05-0.88 (m, 38H), 0.78-0.73 (m, 8H), 0.73-0.66 (m, 8H), 0.65-0.59 (m, 12H).

^{13}C NMR (125 MHz, $CDCl_3$) δ 182.82, 162.32, 157.86, 149.89, 148.98, 144.42, 143.31, 137.07, 131.38, 123.92, 120.90, 112.27, 70.26, 54.40, 43.71, 35.76, 34.78, 34.60, 32.11, 29.87, 29.03, 27.79, 26.50, 23.24, 23.12, 14.58, 11.10.

MS (MALDI-TOF): Calcd for $C_{70}H_{102}O_4S_4$ (M^+): 1134.67, Found: 1135.81.

6,6'-(2,5-Bis(octyloxy)-1,4-phenylene)bis(4,4-bis(2-ethylhexyl)-4H-cyclopenta[1,2-b:5,4-b'] dithiophene-2-carbaldehyde) (5b)

To a solution of 4b (282 mg, 0.25 mmol) in 30 mL THF (tetrahydrofuran), *n*-BuLi (0.62 mL, 2.4 M, 1.5 mmol) was added dropwise under the protection of nitrogen at -78 °C. After the mixture was stirred for 1 h at this temperature, DMF (1 mL) was added in one portion. Then the mixture was warmed to room temperature and stirred overnight. The mixture was poured into water, extracted by ethyl acetate, evaporated to dryness, and the residue was chromatographically

purified on silica gel column eluting with ethyl acetate/petroleum ether (1:15, v/v) to give 5b as an orange oil in a yield of 69% (202 mg).

^{1}H NMR (400 MHz, $CDCl_3$) δ 9.83 (s, 2H), 7.58-7.56 (m, 2H), 7.50-7.48 (m, 2H), 7.24 (d, 2H), 4.17 (t, *J*=6.64 Hz, 4H), 2.04-1.89 (m, 12H), 1.66-1.58 (m, 4H), 1.40-1.31 (m, 8H), 1.08-0.85 (m, 38H), 0.79-0.60 (m, 30H).

^{13}C NMR (125 MHz, $CDCl_3$) δ 182.81, 162.41, 157.86, 149.91, 148.98, 144.46, 143.31, 137.01, 131.25, 123.94, 121.00, 112.39, 70.25, 54.42, 43.72, 35.76, 34.78, 34.61, 32.34, 30.19, 29.92, 29.81, 29.03, 27.96, 27.80, 26.86, 23.24, 14.61, 11.17.

MS (MALDI-TOF): Calcd for $C_{74}H_{110}O_4S_4$ (M^+): 1190.73, Found: 1191.89.

6,6'-(2,5-Bis((2-ethylhexyl)oxy)-1,4-phenylene)bis(4,4-bis(2-ethylhexyl)-4H-cyclopenta[1,2-b:5,4-b']dithiophene-2-carbaldehyde) (5c)

To a solution of 4c (320 mg, 0.28 mmol) in 30 mL THF, *n*-BuLi (0.71 mL, 2.4 M, 1.68 mmol) was added dropwise under the protection of nitrogen at -78 °C. After the mixture was stirred for 1 h at this temperature, DMF (1 mL) was added in one portion. Then the mixture was warmed to room temperature and stirred overnight. The mixture was poured into water, extracted by ethyl acetate, the organic phase was collected and evaporated to dryness, and the residue was chromatographically purified on silica gel column eluting with ethyl acetate/petroleum ether (1:20, v/v) to give 5c as an orange oil in a yield of 87% (290 mg).

^{1}H NMR (600 MHz, $CDCl_3$) δ 9.83 (s, 2H), 7.58-7.56 (m, 2H), 7.50-7.49 (m, 2H), 7.24 (s, 2H), 4.07-4.03 (m, 4H), 2.04-1.89 (m, 8H), 1.67-1.56 (m, 4H), 1.43-1.35 (m, 8H), 1.04-0.91 (m, 46H), 0.79-0.67 (m, 16H), 0.66-0.59 (m, 12H).

^{13}C NMR (125 MHz, $CDCl_3$) δ 182.89, 162.57, 157.86, 150.02, 148.97, 144.68, 143.31, 136.72, 131.39, 123.76, 121.27, 112.20, 72.24, 54.44, 43.77, 40.17, 35.73, 34.71, 34.62, 31.29, 29.70, 29.04, 27.82, 24.63, 23.60, 23.24, 14.53, 11.74, 11.11.

MS (MALDI-TOF): Calcd for $C_{74}H_{110}O_4S_4$ (M^+): 1190.73, Found: 1191.80.

6,6'-(2,5-Dihexyl-1,4-phenylene)bis(4,4-bis(2-ethylhexyl)-4H-cyclopenta[1,2-b:5,4-b'] dithiophene-2-carbaldehyde) (5d)

To a solution of 4d (270 mg, 0.26 mmol) in 30 mL THF (tetrahydrofuran), n-BuLi (0.65 mL, 2.4 M, 1.56 mmol) was added dropwise under the protection of nitrogen at -78 °C. After the mixture was stirred for 1 h at this temperature, DMF (1 mL) was added in one portion. Then the mixture was warmed to room temperature and stirred overnight. The mixture was poured into water, extracted by ethyl acetate, the organic layers were collected and evaporated to dryness. The residue was chromatographically purified on silica gel column eluting with ethyl acetate/petroleum ether (1:20, v/v) to give 5d as an orange oil in a yield of 70% (200 mg).

^{1}H NMR (600 MHz, $CDCl_3$) δ 9.85 (s, 2H), 7.59-7.57 (m, 2H), 7.30 (s, 2H), 6.98-6.97 (m, 2H), 2.80-2.74 (m, 4H), 2.03-1.89 (m, 8H), 1.65-1.57 (m, 4H), 1.38-1.24 (m, 12H), 1.10-0.83 (m, 38H), 0.80-0.73 (m, 12H), 0.71-0.60 (m, 16H).

^{13}C NMR (125 MHz, $CDCl_3$) δ 182.99, 162.41, 157.61, 148.63, 147.47, 143.42, 139.07, 136.40, 134.34, 132.79, 131.38, 122.68, 54.63, 43.81, 35.77, 34.80, 33.64, 32.16, 29.76, 29.25, 29.04, 27.81, 23.35, 23.22, 23.08, 14.60, 11.25.

MS (MALDI-TOF): Calcd for $C_{70}H_{102}O_2S_4$ (M^+): 1102.68, Found: 1103.74.

DOC6-IC

A mixture of 5a (180 mg, 0.16 mmol) and 6a (462 mg, 2.4 mmol) in chloroform was carefully degassed before pyridine was added. After refluxed under N_2 overnight, the reaction was cooled to room temperature. The solvent was evaporated and the residue was purified on silica gel column eluting with DCM/petroleum ether (2:1, v/v) to give DOC6-IC as a dark purple solid in a yield of 93% (220 mg).

^{1}H NMR (500 MHz, $CDCl_3$) δ 8.93 (s, 2H), 8.68 (d, *J*=6.12 Hz, 2H), 7.90 (d, *J*=5.16 Hz, 2H), 7.76-7.69 (m, 4H), 7.65 (s, 2H), 7.52 (s, 2H), 7.30 (s, 2H), 4.21-4.19 (m, 4H), 2.06-1.96 (m, 12H), 1.67-1.60 (m, 4H), 1.50-1.40 (m, 8H), 1.02-0.89 (m, 38H), 0.77-0.71 (m, 16H), 0.67-0.63 (m, 12H).

^{13}C NMR (125 MHz, $CDCl_3$) δ 188.66, 164.96, 160.84, 159.44, 158.81, 149.76, 147.56, 140.02, 137.82, 137.78, 136.89, 134.67, 133.97, 125.06, 123.34, 120.46, 115.29, 111.42, 69.95, 67.00, 53.80, 43.39, 43.32, 35.45, 34.25, 34.02, 31.62, 29.36, 28.52, 28.48, 27.50, 27.29, 26.02, 22.80, 22.69, 14.12, 14.09, 10.63.

MS (MALDI-TOF): Calcd for $C_{94}H_{110}N_4O_4S_4$ (M^+): 1486.74, Found: 1487.26.

DOC8-IC

A mixture of 5b (180 mg, 0.15 mmol) and 6a (494 mg, 2.3 mmol) in chloroform was carefully degassed before pyridine was added. As the same of above reaction and the residue was purified on silica gel column eluting with DCM/petroleum ether (3:2, v/v) to give DOC8-IC as dark purple solid in a yield of 71% (185 mg).

^{1}H NMR (500 MHz, $CDCl_3$) δ 8.93 (s, 2H), 8.68 (d, *J*=6.08 Hz, 2H), 7.90 (d, *J*=5.12 Hz, 2H), 7.75-7.69 (m, 4H), 7.65 (s, 2H), 7.53 (s, 2H), 7.29 (s, 2H), 4.22-4.19 (m, 4H), 2.06-1.95 (m,12H), 1.66-1.58 (m, 4H), 1.52-1.45 (m, 4H), 1.43-1.31 (m, 12H), 1.02-0.89 (m, 38H), 0.77-0.71 (m, 16H), 0.67-0.62 (m, 12H).

^{13}C NMR (125 MHz, $CDCl_3$) δ 188.65, 164.98, 160.79, 159.14, 158.85, 149.75, 147.58, 140.02, 138.91, 138.05, 137.91, 136.89, 134.66, 133.98, 125.06, 123.78, 123.33, 120.48, 119.77, 115.29, 111.41, 69.95, 67.00, 53.82, 43.40, 35.45, 34.25, 34.02, 31.90, 29.71, 29.43, 29.38, 28.52, 27.50, 27.29, 26.39, 22.80, 22.72, 14.16, 14.09, 10.64.

MS (MALDI-TOF): Calcd for $C_{98}H_{118}N_4O_4S_4$ (M^+): 1542.80, Found: 1543.31.

DOC2C6-IC

A mixture of 5c (290 mg, 0.24 mmol) and 6a (708 mg, 3.6 mmol) in chloroform was carefully degassed before pyridine was added. Following the same procedure as the above reaction, DOC2C6-IC was obtained as dark purple solids in a yield of 67% (250 mg).

^{1}H NMR (500 MHz, $CDCl_3$) δ 8.93 (s, 2H), 8.68 (d, *J*=6.04 Hz, 2H), 7.91 (d, *J*=6.40 Hz, 2H), 2H, 7.75-7.69 (m, 4H), 7.65 (s, 2H), 7.54 (s, 2H), 7.29 (s,2H), 4.14-4.08 (m, 4H), 2.05-1.92 (m,8H), 1.72-1.60 (m, 4H), 1.46-1.35 (m, 8H), 1.05-0.91 (m, 46H), 0.79-0.69 (m, 16H), 0.67-0.60 (m, 12H).

^{13}C NMR (125 MHz, $CDCl_3$) δ 188.69, 165.13, 160.78, 159.13, 158.81, 149.85, 147.76, 140.03, 138.92, 137.81, 136.89, 134.68, 133.99, 125.07, 123.67, 123.34, 120.75, 119.78, 115.28, 111.34,

71.84, 67.03, 53.82, 43.38, 39.63, 35.47, 34.20, 34.02, 30.81, 29.72, 29.21, 28.52, 28.46, 27.46, 27.31, 24.23, 23.12, 22.80, 14.19, 14.08, 11.26, 10.65.

MS (MALDI-TOF): Calcd for $C_{98}H_{118}N_4O_4S_4$ (M^+): 1542.80, Found: 1542.75.

DC6-IC

A mixture of 5d (200 mg, 0.18 mmol) and 6a (527 mg, 2.7 mmol) in chloroform was carefully degassed before pyridine was added. Following the same procedure as the above reaction, DC6-IC was obtained as dark purple solids in a yield of 57% (150 mg).

^{1}H NMR (500 MHz, $CDCl_3$) δ 8.95 (s, 2H), 8.69 (d, *J*=6.12 Hz, 2H), 7.91 (d, *J*=6.36 Hz, 2H), 7.76-7.65 (m, 6H), 7.32 (s, 2H), 7.04-7.03 (m, 2H), 2.81-2.78 (m, 4H), 2.07-1.93 (m, 8H), 1.65-1.59 (m, 4H), 1.38-1.26 (m, 12H), 1.11-0.85 (m, 38H), 0.80-0.70 (m, 16H), 0.69-0.63 (m, 12H).

^{13}C NMR (125 MHz, $CDCl_3$) δ 188.61, 164.97, 160.82, 158.72, 158.16, 150.60, 139.98, 138.68, 138.36, 138.01, 136.86, 134.76, 134.10, 133.95, 132.23, 125.13, 123.45, 122.61, 120.13, 115.15, 67.50, 54.14, 43.51, 43.51, 43.23, 35.51, 34.24, 33.24, 31.70, 31.46, 29.72, 29.29, 28.76, 28.47, 27.34, 22.91, 22.79, 22.62, 14.09, 10.78, 10.51.

MS (MALDI-TOF): Calcd for $C_{94}H_{110}N_4O_4S_4$ (M^+): 1454.75, Found: 1454.84.

DOC2C6-2F

A mixture of 5a (200 mg, 0.17 mmol) and 6b (210 mg, 0.91 mmol) in chloroform was carefully degassed before pyridine was added. After refluxed under N_2 at room temperature overnight, the solvent was evaporated and the residue was purified on silica gel column eluting with DCM/petroleum ether (4:1, v/v) to give DOC2C6-2F as a dark purple solid in a yield of 63% (170 mg).

^{1}H NMR (500 MHz, $CDCl_3$) δ 8.90 (s, 2H), 8.54-8.51 (m, 2H), 7.67-7.64 (m, 4H), 7.56-7.55 (m, 2H), 7.30 (s, 2H), 4.11-4.10 (m, 4H), 2.05-1.92 (m, 8H), 1.72-1.60 (m, 4H), 1.46-1.35 (m, 8H), 1.05-0.91 (m, 46H), 0.79-0.72 (m, 16H), 0.67-0.62 (m, 12H).

^{13}C NMR (125 MHz, $CDCl_3$) δ 186.39, 166.99, 160.11, 159.98, 158.73, 155.38, 153.31, 150.06, 148.65, 138.99, 138.49, 138.17, 137.92, 136.70, 134.54, 123.88, 121.04, 119.00, 115.05, 114.88, 112.49, 111.48, 72.02, 67.54, 54.00, 43.50, 39.74, 35.64, 34.33, 34.15, 30.93, 29.34, 28.58, 27.59, 27.44, 24.36, 23.25, 22.93, 14.32, 14.22, 11.39, 10.75.

MS (MALDI-TOF): Calcd for $C_{98}H_{118}N_4O_4S_4$ (M^+): 1614.77, Found: 1614.32.

Supplementary References

1. Hassanien AS., *et al.* Influence of composition on optical and dispersion parameters of thermally evaporated non-crystalline Cd 50 S 50−x Se x thin films. *J. Alloy. Compd.* **648**, 280-290 (2015).
2. Ma D., *et al.* The design of highly efficient polymer solar cells with outstanding short-circuit current density based on small band gap electron acceptor. *Dyes Pigm.***150**, 363-369 (2018).
3. Comyn J. Contact angles and adhesive bonding. *Int. J. Adhes. Adhes.* **12**, 145-149 (1992).
4. Nilsson S., *et al*. Morphology and phase segregation of spin-casted films of polyfluorene/PCBM blends. *Macromolecules* **40**, 8291-8301 (2007).
5. Alexander H*., et al.* A SAXS/WAXS/GISAXS beamline with multilayer monochromator. *J. Phys.: Conf. Ser*. **247**, 012007 (2010).
